# Editorial: Invertebrate UDP-Glycosyltransferases: Nomenclature, Diversity and Functions

**DOI:** 10.3389/fphys.2021.748290

**Published:** 2021-09-06

**Authors:** Seung-Joon Ahn, Thomas Chertemps, Martine Maïbèche, Steven J. Marygold, Thomas Van Leeuwen

**Affiliations:** ^1^Department of Biochemistry, Molecular Biology, Entomology and Plant Pathology, Mississippi State University, Starkville, MS, United States; ^2^Sorbonne Université, INRA, CNRS, IRD, UPEC, Institut d'Ecologie et des Sciences de l'Environnement de Paris, Paris, France; ^3^FlyBase, Department of Physiology, Development and Neuroscience, University of Cambridge, Cambridge, United Kingdom; ^4^Laboratory of Agrozoology, Department of Plants and Crops, Faculty of Bioscience Engineering, Ghent University, Ghent, Belgium

**Keywords:** UDP-glycosyltransferase, multigene family, invertebrate, detoxification, homeostasis

Glycoside conjugation is one of the important mechanisms in xenobiotic detoxification in living organisms (Smith, 1955). It increases water solubility making the compounds more easily excretable, thereby protecting the cellular system from damage by toxic compounds, well-known as an important component of Phase II detoxification in human (Meech et al., 2019). Plants can conjugate growth hormones, pigments, and secondary metabolites, maintaining homeostasis in their growth and physiological functions (Bowles et al., 2006). Macrolide antibiotics such as vancomycin are synthesized through glycoside conjugation in some bacteria (Quirós et al., 2000). However, little is known about the glycoside conjugation in invertebrates, a highly diverse group of animals. Recent work on chemo-ecological interactions among different living organisms and environments, such as arthropod-plant interactions, chemical arms-races, and pesticide resistance, have revisited the significance of glycoside conjugation from ecological roles to molecular functions (Heckel, 2018).

The conjugation of sugar molecules to small lipophilic compounds is mediated by a superfamily of UDP-glycosyltransferases (UGTs) found in all kingdoms of life, sharing a conserved signature motif in their protein sequences (Bock, 2016). UGTs catalyze the conjugation of an activated sugar form, uridine diphosphate (UDP)-glycoside, with a variety of small compounds, such as plant secondary metabolites, insect molting hormones, pesticides, and other xenobiotics. Thanks to the easy access to genome sequencing technology, a wave of generated genomic sequence data has facilitated extensive searches of the UGT superfamily in diverse groups of invertebrates, revealing a large number of UGT genes in each species (Ahn et al., 2012). In addition, various transcriptome analyses have shown multifaceted expression profiles in relation to tissues, hosts, chemicals, or other environmental factors suggesting novel associated functions (Bozzolan et al., 2014; Younus et al., 2014; Snoeck et al., 2019; Pan et al., 2020). However, the exponential rise in the number of invertebrate UGT genes necessitates the use of a universal nomenclature system to enable systematic identification and interspecies comparisons of this highly-diverse multigene family. Also, as the UGT-related studies have so far most frequently focused on arthropods including insects, a taxonomic extension beyond arthropods would be desirable.

In this Research Topic, we introduce four original research articles covering a broad scope of organisms including two insects (Arthropoda), one parasitic nematode (Nematoda), and one coral (Cnidaria) species. Ahn and Marygold present a comprehensive example of UGT nomenclature practice in the fruit fly, *Drosophila melanogaster*, though it can be used as a standard reference for Diptera (flies and mosquitoes) and other insects. For the naming, the authors took advantage of a wealth of information, such as genomic orientation, phylogenetic analysis, as well as gene structure of all UGT sequences. A comparative analysis of the UGT gene family among 19 *Drosophila* species is also presented, illustrating how systematic nomenclature facilitates the comparison of this multigene family among different species. A brief summary of the *Drosophila* UGT functions studied thus far provides a glimpse of the current status of this Research Topic in this model insect.

As a good example of the role of UGT in insect-plant interactions, Israni et al., show that the fall armyworm, *Spodoptera frugiperda*, detoxifies plant defense compounds from grass crops using its UGT enzyme. The researchers confirmed that glucosylation plays a major role in benzoxazinoids (BXDs) metabolism by analyzing the ingested BXDs, and further explored biochemical activities of UGTs using heterologous expression and RNAi. They conclude that a single UGT enzyme is likely responsible for detoxification of BXD, thereby allowing the herbivore to thrive in the crop plants.

Kellerová et al., demonstrate that UGTs take part in biotransformation of an anthelmintic drug, albendazole, in the nematode *Haemonchus contortus*, a common parasite of ruminants. The free-living juvenile stages can easily get into contact with the anthelmintics on pasture. This study shows that *H. contortus* juveniles exhibit dynamic expression of UGT genes that is affected by treatment with and/or susceptibility to the drug. This work adds a precious example of UGT roles in nematodes, which have received relatively little attention to date.

Finally, Rougée et al., report a chronic effect of the xenoestrogen, 4-nonylphenol, on UGT activity in the reef building coral, *Pocillopora damicorniscoral*. UGT activity in this marine cnidarian species usually fluctuates with lunar cycle, but remains at a consistently high level when exposed to 4-nonylphenol for long periods of time. This suggests that chronic environmental exposure to the pollutant causes significant dysregulation in xenobiotic detoxification and homeostasis in marine invertebrates.

Taken together, the articles published here discuss the diversity and versatility of invertebrate UGT functions in a broad range of circumstances, providing the impetus and opportunity for further in-depth study. Not only should the metabolic detoxification be further investigated, but also other non-canonical UGT functions are expected to be revealed from invertebrates that have evolved into a large variety of forms. We believe these studies help to draw more attention to this multifaceted enzyme family.

## Author Contributions

All authors listed have made a substantial, direct and intellectual contribution to the work, and approved it for publication.

## Conflict of Interest

The authors declare that the research was conducted in the absence of any commercial or financial relationships that could be construed as a potential conflict of interest.

## Publisher's Note

All claims expressed in this article are solely those of the authors and do not necessarily represent those of their affiliated organizations, or those of the publisher, the editors and the reviewers. Any product that may be evaluated in this article, or claim that may be made by its manufacturer, is not guaranteed or endorsed by the publisher.
